# Regulation of Tlx3 by Pax6 is required for the restricted expression of Chrnα3 in Cerebellar Granule Neuron progenitors during development

**DOI:** 10.1038/srep30337

**Published:** 2016-07-25

**Authors:** Thulasi Sheela Divya, Soundararajan Lalitha, Surendran Parvathy, Chandramohan Subashini, Rajendran Sanalkumar, Sivadasan Bindu Dhanesh, Vazhanthodi Abdul Rasheed, Mundackal Sivaraman Divya, Shubha Tole, Jackson James

**Affiliations:** 1 Neuro Stem Cell Biology Laboratory, Neurobiology Division, Rajiv Gandhi Centre for Biotechnology, Thiruvananthapuram, Kerala-695 014, India; 2 Department of Biological Sciences, Tata Institute of Fundamental Research, Mumbai-400005, India

## Abstract

Homeobox gene *Tlx3* is known to promote glutamatergic differentiation and is expressed in post-mitotic neurons of CNS. Contrary to this here, we discovered that Tlx3 is expressed in the proliferating progenitors of the external granule layer in the cerebellum, and examined factors that regulate this expression. Using Pax6^−/−^Sey mouse model and molecular interaction studies we demonstrate Pax6 is a key activator of Tlx3 specifically in cerebellum, and induces its expression starting at embryonic day (E)15. By Postnatal day (PN)7, Tlx3 is expressed in a highly restricted manner in the cerebellar granule neurons of the posterior cerebellar lobes, where it is required for the restricted expression of nicotinic cholinergic receptor-α3 subunit (Chrnα3) and other genes involved in formation of synaptic connections and neuronal migration. These results demonstrate a novel role for Tlx3 and indicate that Pax6-Tlx3 expression and interaction is part of a region specific regulatory network in cerebellum and its deregulation during development could possibly lead to Autistic spectral disorders (ASD).

Tlx3 also known as Hox11L2 or Rnx is a homeo-box transcription factor that is identified to be expressed specifically in spinal cord motor neurons, brain stem and cerebellum implicating a tight regulation only in particular *niche* of the nervous system^1^. In the spinal cord, it is reported to be expressed only in post-mitotic neural progenitors and is responsible for instructing a glutamatergic neuronal fate by suppressing GABAergic fate specifying factors such as Lbx1 and Pax2^1^,^2^. Other reports have demonstrated that the GABAergic determining factor Ptf1α can repress Tlx3 expression in the spinal cord through Prdm13, thereby promoting a GABAergic fate^3^. It is also known that Tlx3 KO mice die immediately after birth due to excessive GABAergic inputs and central hypoventilation caused due to improper development of medulla^4^. Expression of Tlx3 is also critical for the generation of first order relay sensory neurons and expression of specific cholinergic peptides during mouse sympathetic neuron development^5^,^6^. Further, Brn3a and Drg11 are shown to be target genes of Tlx3 during development and specification of dorsal horn neuron subtypes. Even transcription factors such as Islet1, EBF2 and Phox2a are determined to be highly dependent on Tlx3 expression during neural development^7^.

Although the role of Tlx3 in excitatory versus inhibitory neural fate specification is established in the spinal cord, the actual mechanism of its regulation or downstream functions is still obscure in the other regions. In this study, we were particularly interested in understanding the regulation of Tlx3 in cerebellum since our data and others have shown Tlx3 expression to be limited only to the posterior lobes of the cerebellum. These results raise an interesting question regarding the restricted expression of Tlx3 within the cerebellum and its functional implication to the posterior lobes. Morphologically, both the anterior and posterior lobes are similar but functionally they are different, and this difference in function could be attributed to the differential expression of specific genes such as Tlx3 in distinct areas of the cerebellum. Therefore, to understand this regulation of Tlx3 in cerebellum, we first need to know the upstream regulators of Tlx3 and its downstream effectors.

Previous reports have shown that proneural gene Mash-1 and ubiquitous transcription factor NFY can activate Tlx3 in spinal cord and neuroblastoma cell lines respectively^8^,^9^. Other reports in mesenchymal stem cells have shown the role of Wnt signaling in activating Tlx3 and subsequent neuronal differentiation^10^. Although Mash-1, NFY and Wnt signaling are the known activators of Tlx3 in other cell types, we did not take them into account in the current study and instead explored the possibility Pax6 acting as an activator of Tlx3. The reason for precisely selecting Pax6 as an activator of Tlx3 is its evident expression in glutamatergic cerebellar granule neurons (CGNs) during early development^11^ and also the presence of Pax6 binding sites in the active proximal promoter region of *Tlx3.* Here, we provide convincing evidence for the involvement of Pax6 in activating Tlx3 in proliferating CGNs of posterior cerebellar lobes, which in turn promotes the particular expression of nicotinic cholinergic receptor α3 subunit (Chrnα3) and other genes involved in formation of synaptic connections and neuronal migration during development. To our knowledge this is the first report where we have demonstrated a new role for Tlx3 in promoting the expression of genes involved in formation of synaptic connections, neuronal migration and compartmentalized expression of Chrnα3 only in the posterior lobes of developing cerebellum. Moreover, this study gains importance as it points to the involvement of Pax6-Tlx3 regulatory network connected to Chrnα3 expression and possibly to other genes involved in synaptic connection formation and neuronal migration which is shown to be reduced in cerebellum of patients with ASD^12^.

## Results

### Tlx3 expression is restricted to posterior lobes of developing cerebellum

**Figure 1:**
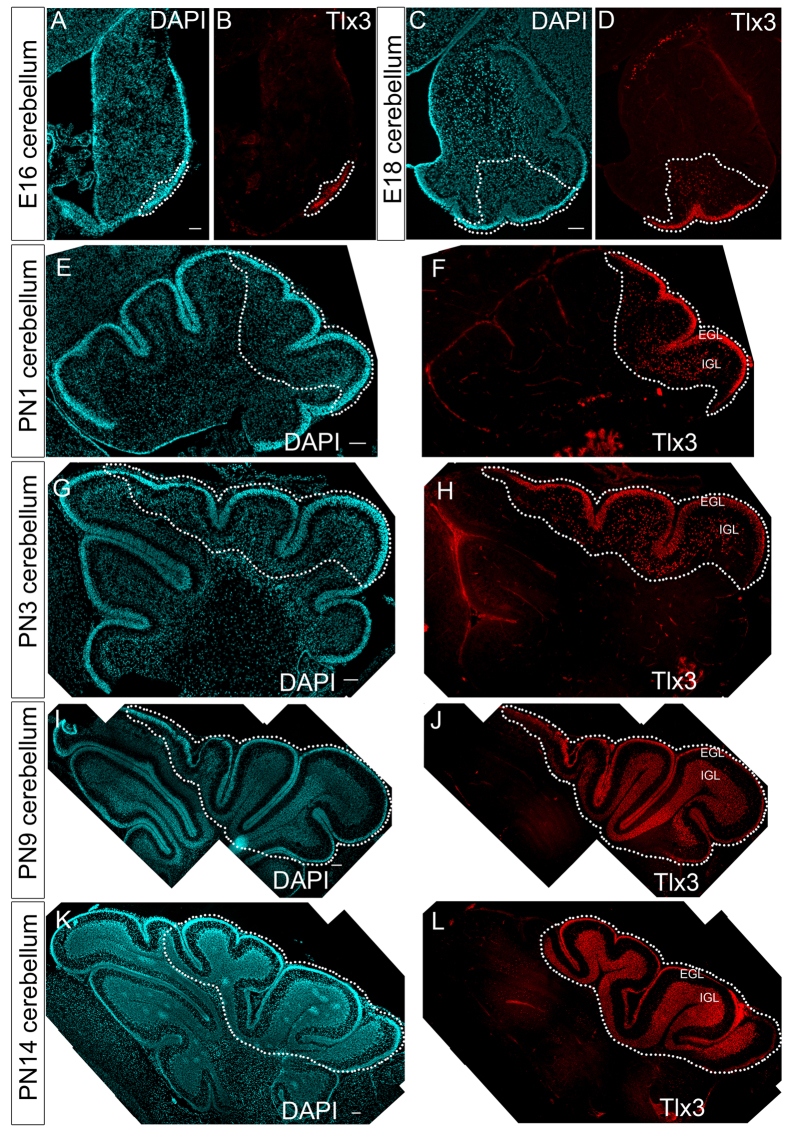
Tlx3 expression is restricted to the posterior lobes of developing cerebellum. Immunohistochemical analysis of Tlx3 in E16 (**A**,**B**), E18 (**C**,**D**) PN1 (**E**,**F**), PN3 (**G**,**H**), PN9 (**I**,**J**) and PN14 (**K**,**L**) confirmed that Tlx3 expression is restricted specifically to the posterior lobes of cerebellum. Image (**A–L**) is generated by stitching together multiple images using Photoshop software. Scale bar = 100 μm.

In order to understand the expression pattern of Tlx3 in cerebellum, we carried out immunofluorescence analysis in developing cerebellum of E16-PN14 embryos. During early embryogenesis the CGN progenitors migrate from the rhombic lip tangentially along the cerebellar surface to create a second germinative zone known as the External Granule Layer (EGL). Our results show the initial expression of Tlx3 by E16 stage in a small group of progenitors restricted to the posterior EGL (Fig. 1A,B). This group of progenitors expressing Tlx3 later spread out as a streak along the posterior EGL by E18 stage (Fig. 1C,D). Further by PN1, these progenitors expand and populate the EGL of the posterior lobes of the cerebellum (Fig. 1E,F), which then enter differentiation and move radially inward to form the internal granule layer (IGL) during late embryonic and postnatal stages (Fig. 1G–L)^13^. This restricted expression of Tlx3 confined only to the EGL cells of the posterior lobes prompted us to further look into the factors that could induce the expression of Tlx3.

### Pax6 induces Tlx3 expression in cerebellum

**Figure 2:**
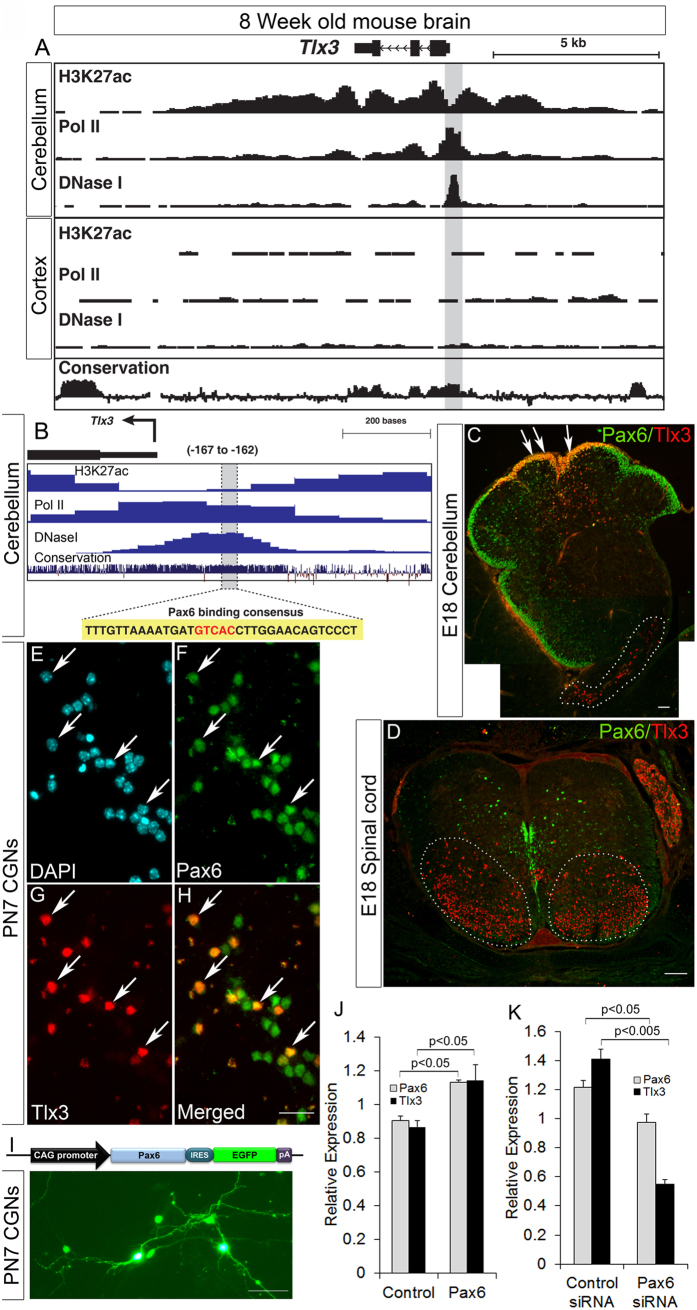
Pax6 regulate Tlx3 expression in cerebellar granule neurons. (**A**) ENCODE profiles for lysine 27 acetylation at Histone H3(H3K27ac), DNase I hypersensitive region (DNase I) and Polymerase II occupancy (Pol II) at the *Tlx3* locus in 8 week mouse cerebellum and cortex. The significant PolII/DNaseI peak at the proximal promoter region in the cerebellum refers to an open chromatin region which assembles the active transcriptional complex. (**B**) Expanded view of the open chromatin at the proximal Tlx3 promoter region in Cerebellum showing Pax6 binding sites. (**C**) Immunohistochemical analysis of E18 cerebellum where Pax6 and Tlx3 co-express specifically in the CGNs of the posterior lobes only and not in the anterior lobes. (**D**) Immunohistochemical analysis in the spinal cord showed that Pax6 and Tlx3 are not co-expressed. (**E**–**H**) Immunocytochemical analysis of Pax6 and Tlx3 in *in vitro* cultured cerebellar granule neurons showed that all Tlx3 active cells (~50%) co-expressed Pax6. (**I**) Schematic of Pax6 expression construct. Cerebellar granule neurons electroporated with this construct express EGFP and confirm Pax6 overexpression. (**J**) Real-time PCR analysis showed that Tlx3 is significantly up-regulated (p < 0.05) by Pax6 overexpression. (**K**) Down regulation of Pax6 using siRNA significantly down-regulated Pax6 (p < 0.05) and correspondingly Tlx3 (p < 0.005) expression in cerebellar granule neurons. Image E is generated by stitching together multiple images using Photoshop software. Data are expressed as Mean ± S.D. of triplicates (*n* = 3) from three different experiments. Scale bar D,E = 100 μm, F–I = 25 μm and J = 50 μm.

In order to examine the factors that could regulate Tlx3 gene expression, we mined publically available datasets for active chromatin attributes in the cerebellum and compared with cortex wherein Tlx3 is not expressed. The data sets for open chromatin configuration (DNaseI seq), polymerase II occupancy and H3K27ac histone mark are indicative of active promoter and/or regulatory regions and were extracted from ENCODE (http://genome.ucsc.edu/ENCODE/)^14^. By comparing the profiles in the *Tlx3* locus, it is evident that the active chromatin attributes were selectively enriched in the cerebellum compared to cortex (Fig. 2A). The profiles indicate the presence of an active/open region in the proximal promoter; this region could potentially be assembling the active transcriptional complex. By analyzing the active promoter sequence for transcription factor binding using JASPAR and ALGGEN-PROMO, several transcription factors including Pax6 emerged. As Pax6 is expressed in the select cerebellar regions and have binding sites in the active proximal promoter region, we reasoned that Pax6 could be a potential activator of *Tlx3* (Fig. 2B). To test this, we selected HeLa cells that had constitutive expression of Tlx3 to carry out initial *in vitro* interaction studies of Pax6 with Tlx3. Expression of Tlx3 was confirmed with expression of EGFP driven by *Tlx3* promoter (Fig. S1A). We also confirmed the expression of Pax6 in HeLa cells (Fig. S1B). HeLa cells were further transfected with Pax6 and dominant negative Pax6 (Pax6Δ286, a kind gift from Dr Elizabeth Fini) expressing plasmids along with Tlx3 promoter and luciferase assay was carried to confirm the interaction. Our results showed that overexpression of Pax6 significantly enhanced (p < 0.05) Tlx3 promoter activity whereas transfection of dnPax6 significantly reduced (p < 0.05) the activation of Tlx3 (Fig. S1C). Further real-time RT-PCR analysis confirmed the Pax6 mediated Tlx3 regulation (Fig. S1D,E). To further confirm whether there is any possible binding of Pax6 on Tlx3 promoter we mutated the core sequence of the Pax6 binding site and cloned the mutated Tlx3 promoter into pGl3 basic vector (Fig. S1F). Initial bioinformatics analysis has revealed the presence of a conserved Pax6 binding site in the proximal promoter region of Tlx3. Luciferase analysis was carried out using wild type as well as mutated Tlx3 luciferase construct which demonstrated a significant reduction (p < 0.05) in Tlx3 expression with mutated construct compared to Wt-Tlx3 construct (Fig. S1G). Over expression of Pax6 along with Tlx3-Luc construct significantly enhanced (p < 0.005) the promoter activity but it was significantly reduced (p < 0.005) when Pax6 was transfected along with mutated mTlx3-Luc construct (Fig. S1G). These results demonstrate that the mutated region in the Tlx3 promoter could be a possible binding site for Pax6. We assume that the other predicted Pax6 binding sites on Tlx3 promoter, which was less conserved might also be involved with Pax6 interaction and this could be a possible reason for the small up-regulation of luciferase activity observed with mTlx3-Luc+Pax6 construct compared to mTlx3-Luc alone. Therefore, from these preliminary results, it appears that indeed Pax6 can act as an activator of Tlx3.

Since we now know that Pax6 could act as an activator of Tlx3 *in vitro*, we further checked the possibility of recapitulating a similar mechanism *in vivo.* To achieve this, we scanned the known Tlx3 expressing regions such as spinal cord, brain stem and cerebellum of E18 embryos for co-localization of Pax6 and Tlx3^4^. To our surprise we found that Tlx3 and Pax6 were co-localized only in the granule neurons in the cerebellum whereas, Tlx3 did not co-localize with Pax6 in the brain stem and spinal cord (Fig. 2C,D). It is to be noted that Pax6 marks all the glutamatergic progenitors in developing cerebellum, and Tlx3 is also known to induce glutamatergic neuronal differentiation^2^,^15^. Therefore, these observations show a very interesting correlation between the two genes and hint at the possibility of Pax6 mediated Tlx3 regulation during cerebellar granule neuron development. This notion is supported further by the fact that Pax6 KO shows deficiency in cerebellum development especially the granule neurons^16^. Therefore from our preliminary Pax6-Tlx3 regulation studies and the above reports there is very high potential for Pax6 to be the specific activator of Tlx3 in cerebellum during development.

Since, now we know that Pax6 and Tlx3 are co-expressed in cerebellum, we went ahead and used PN7 cerebellar granule neuron culture as a suitable system to confirm our results. The cultured CGNs were initially characterized using neural and glutamatergic markers such as β-III Tubulin, vGlut1 and GABAα6 that had a significantly high expression in these cells (Fig. S2A–P). Further characterization with Pax6 and Tlx3 antibody showed that all the Tlx3 positive cells were also positive (100%) for Pax6 (Fig. 2E–H). Real-time RT-PCR analysis was carried out further to evaluate the regulation of Tlx3 by Pax6 using cDNA obtained from CGN cultures after transfection with Pax6 expressing construct (Fig. 2I). Our results demonstrated that Pax6 overexpression significantly up-regulated Tlx3 expression (Fig. 2J). To further confirm this we used Pax6 siRNA to check whether blocking Pax6 affected Tlx3 expression in CGNs. For this different concentration of Pax6 siRNA were used, and the concentration of 25 picomoles was selected for analysis since it effectively blocked Pax6 expression. Real-time RT-PCR analysis corroborated the above results and proved that down-regulation of Pax6 in turn suppressed Tlx3 expression in CGNs (Fig. 2K). The specificity of Pax6 siRNA was confirmed by checking the down regulation of Pax6 and corresponding down regulation of Tlx3 by real-time RT-PCR analysis at different time intervals (24 h, 36 h and 60 h) after transfection. Our results showed a drastic reduction in Pax6 expression and corresponding Tlx3 expression by 36 h after Pax6 siRNA transfection (Fig. S2Q). Both Pax6 and Tlx3 expression pattern showed a reversing trend by 60 h after transfection. These perturbation studies further strengthened our hypothesis that Pax6 can act as a positive regulator of Tlx3 in cerebellum.

### Pax6 induces expression of Tlx3 in CGNs of posterior lobe of developing cerebellum

**Figure 3:**
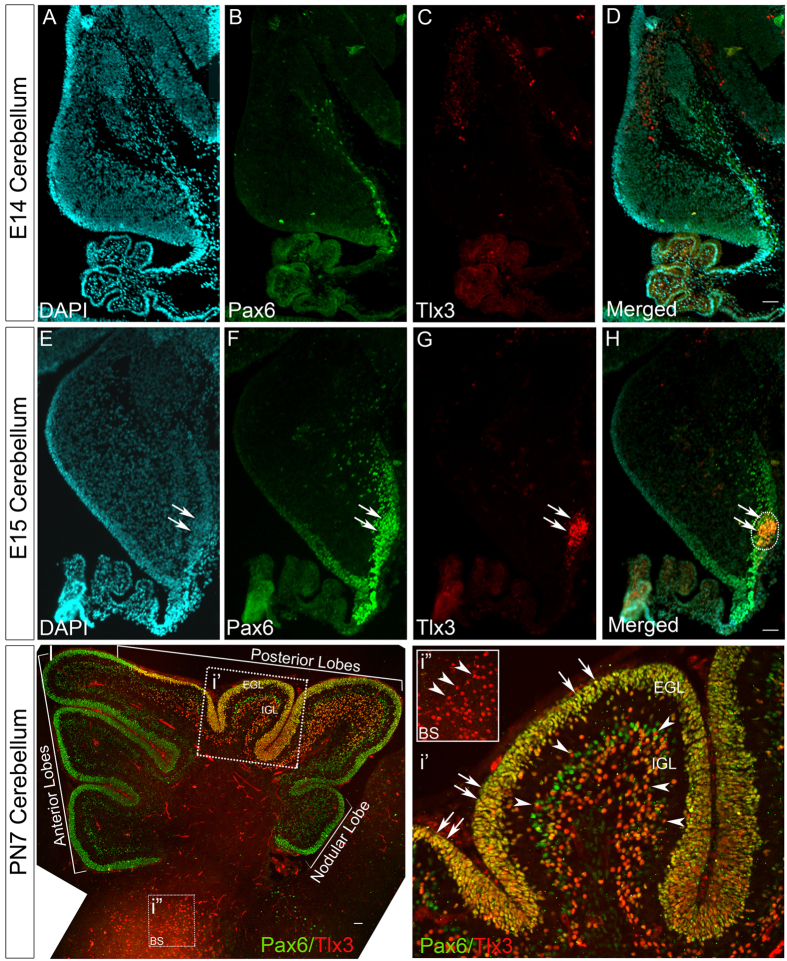
Tlx3 expression is initiated in a group of Pax6 positive progenitors at E15 stage and later gets restricted to posterior lobes of the cerebellum. (**A**–**D**) Immunohistochemical analysis of E14 cerebellum show the initial migration of Pax6 positive CGNs from Rhombic lip. (**E**–**H**) Tlx3 expression is initiated in a small group of Pax6 positive migrating progenitors by E15 stage (indicated by arrows). (**I**) Tlx3 expression is restricted specifically to the posterior lobes of the cerebellum by PN7 stage and these progenitors also co-express Pax6. (i’) Magnified area of the cerebellum showing co-expression of Tlx3 and Pax6 in EGL (arrows) and IGL (arrowheads) of the cerebellum. In addition to this, we do see Pax6 positive progenitors which are negative for Tlx3. (i”) Magnified area of brainstem showing Tlx3 active cells devoid of Pax6 expression. Image I is generated by stitching together multiple images using Photoshop software. Scale bar = 100 μm.

Since, our results have proved that Pax6 can act as a positive regulator of Tlx3, we next wanted to identify the stage and pattern of Pax6 mediated Tlx3 expression during development. To further understand the expression pattern of Pax6 and Tlx3 in cerebellum, we did a co-immunofluorescence analysis in cerebellum at different developmental stages. Here, we did not find any Tlx3 expression in E14 embryonic cerebellum even though the Pax6 positive CGNs have started developing in the EGL (Fig. 3A–D). Interestingly by E15 stage the Tlx3 expression starts as a streak in EGL especially in the posterior region and totally co-localizes with Pax6 (Fig. 3E–H). Since we already know that all Tlx3 active cells are also positive for Pax6, it further confirms that Pax6 is required as an activator for Tlx3 expression.

In order to further analyze the expression pattern of Pax6 and Tlx3 in later stages of development, immunohistochemical analysis of postnatal day 7 (PN7) mouse cerebellums were carried out. PN7 is the stage where cerebellar granule neurons undergo maximum proliferation and the deep fissures separating the lobes starts developing^17^. Our results showed that the expression of Tlx3 was very specific to CGNs in the posterior lobes (starting just after the primary fissure) of the cerebellum and co-expressed with Pax6. Expression of both Pax6 and Tlx3 pertained even in the CGNs that have migrated to the IGL (Fig. 3i’).

We also observed few cells within the IGL that were Pax6^+ve^ and Tlx3^−ve^ (Fig. 3i’). Previous reports have shown Pax6^+ve^ unipolar brush cells (UBCs) in the IGL^15^. To check whether these Pax6^+ve^ and Tlx3^−ve^ cells are UBCs we carried out a co-immunofluorescence analysis with Tlx3 and Tbr2 that labels UBCs. Our results showed that none of the Tbr2^+ve^ cells co-localized with Tlx3 in the IGL confirming that these Pax6^+ve^ and Tlx3^−ve^ neurons are indeed UBCs (Fig. S3A–D). It was also interesting to note that the CGNs in the anterior and nodular lobes expressed only Pax6 and were devoid of Tlx3 expression (Fig. 3I). We also observed that the Tlx3 expressing progenitors in the brain stem were devoid of Pax6 expression (Fig. 3i”) which further demonstrates the specific interaction of Pax6 with Tlx3 only in the cerebellum.

### Tlx3 expression is down regulated in Pax6^−/−^ Sey mouse cerebellum but unaffected in spinal cord

**Figure 4:**
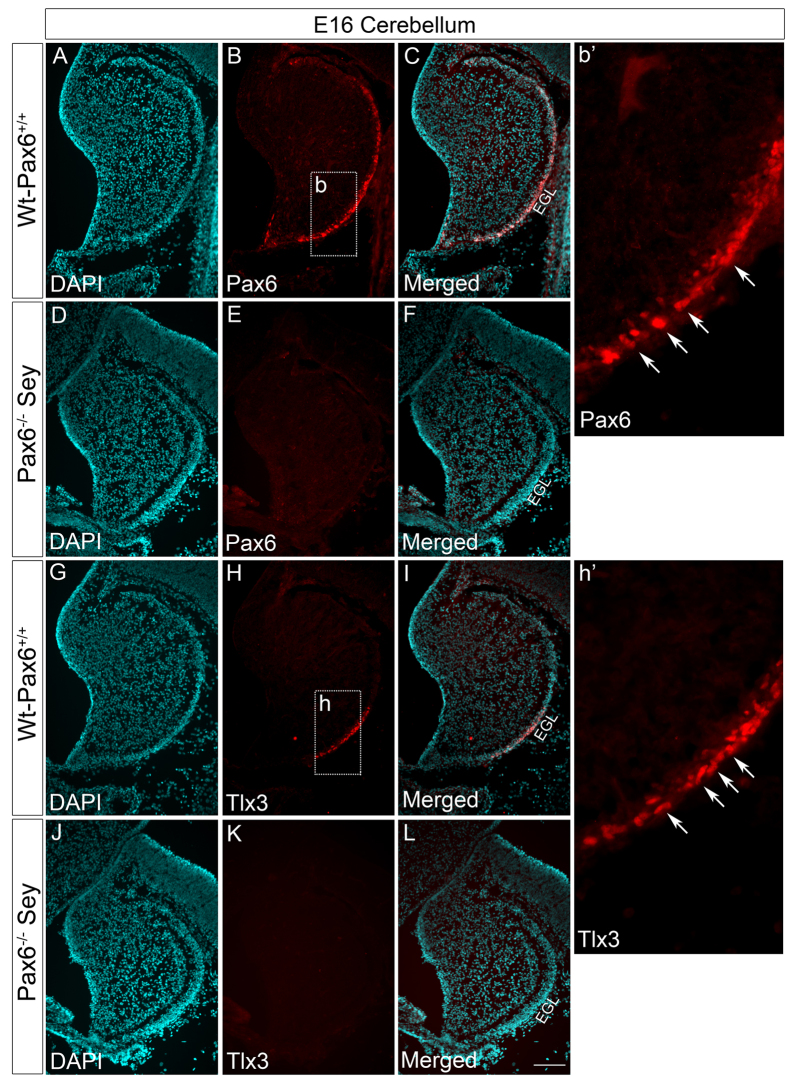
Tlx3 expression is completely abolished in Pax6^−/−^ Sey cerebellum. (**A–C**) Immunohistochemical analysis of E16 Wt-Pax6^+/+^ cerebellum showed expression of Pax6 in EGL layer. (**b’**) Magnified area of Pax6 positive region in EGL of the cerebellum. (**D**–**F**) Pax6 expression was entirely absent in Pax6^−/−^Sey mouse cerebellum. (**G**–**I**) Immunohistochemical analysis of E16 Wt-Pax6^+/+^ cerebellum showed that Tlx3 expression pertained to EGL of perspective posterior region of the cerebellum. (**h’**) Magnified area of Tlx3 positive region in EGL of cerebellum. (**J**–**L**) Tlx3 expression was entirely absent in Pax6^−/−^Sey mouse cerebellum indicating that Pax6 is critical for expression of Tlx3. Scale bar = 100 μm.

To further check the specificity of Pax6 mediated Tlx3 activation in the developing cerebellum, we looked for Tlx3 expression in E16 Pax6^−/−^ Sey mice. Here, we used E16 embryos since these mutants are lethal by E18 stage, which makes it impossible to analyze the cerebellar development and lobulation defects in postnatal stages. These mice also exhibit developmental deformities in eyes as well as nasal placode^18^. Previous studies in conditional Pax6 KO cerebellum have shown that the lack of Pax6 expression affects the differentiation of cerebellar granule cells, and it’s signaling to the neighboring Purkinje cells^16^. Immunohistochemical analysis with Pax6 antibody showed the expression of Pax6 in control Wt-Pax6^+/+^ cerebellum as a stream in the EGL that migrates from the Rhombic Lip (Fig. 4A–C,b’) but was entirely absent in EGL of Pax6^−/−^ Sey mice as expected (Fig. 4D–F). Tlx3 expression was evident in a group of cells towards the posterior side of EGL and all these cells were co-localized with Pax6 in Wt-Pax6^+/+^ Sey mice (Fig. 4G–I,h’). Contrary to this, Tlx3 expression was entirely reduced in EGL of Pax6^−/−^ Sey mutants (Fig. 4J–L). The specificity of Pax6 mediated Tlx3 expression in the cerebellum was further confirmed by the fact that Tlx3 expression was still observed in the spinal cord of Pax6^−/−^ Sey mutants (Fig. S3E–G). Therefore, other molecular mechanisms may regulate Tlx3 expression in different regions of the developing CNS. These results show that Tlx3 expression is entirely dependent on Pax6 in the developing cerebellum, and its absence clearly affects Tlx3 expression specifically in cerebellar granule neurons. Only a few cells that faintly express Tlx3 persist in the EGL of the E17.5 Pax6^−/−^Sey mouse (data not shown), suggesting some compensatory mechanisms may exist that induces this low level of expression.

### Tlx3 expression starts during the proliferative stage in cerebellar granule neurons and maintains its expression till the neurons become mature

**Figure 5:**
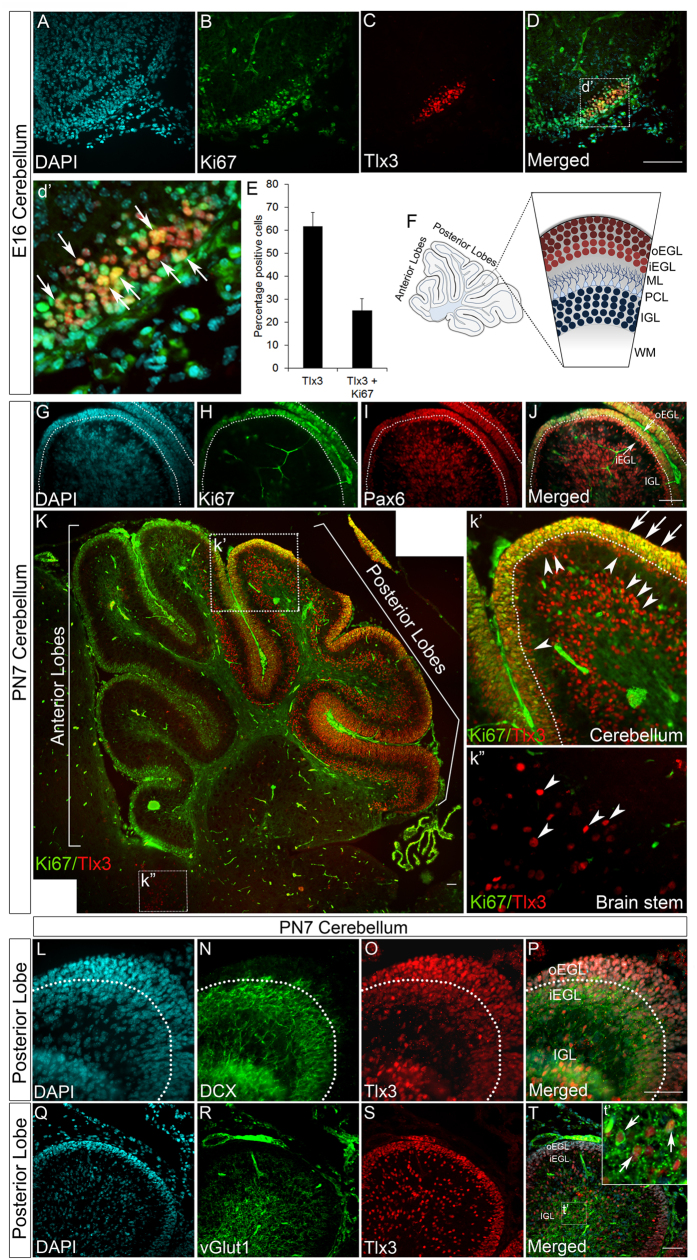
Tlx3 is expressed in proliferating cerebellar granule neurons. (**A**–**D**) Tlx3 positive CGNs of E16 cerebellum co-express Ki67 showing that Tlx3 is expressed in proliferating progenitors. (d’) Magnified region of Tlx3 and Ki67 co-expressing progenitors. (**E**) Graph depicting percentage of cells positive for Tlx3 and Tlx3+Ki67. ~50% of the Tlx3 positive cells co-express Ki67. (**F**) Schematic showing different layers of cells formed in a mature cerebellum that contains proliferating outer EGL (oEGL), post-mitotic inner EGL (iEGL), Molecular layer (ML), Purkinje cell layer (PCL), mature IGL and White Matter (WM). (**G**–**J**) Immunohistochemical analysis with Pax6 and Ki67 in PN7 cerebellum differentiates the oEGL from the iEGL where the oEGL express both Pax6 and Ki67 and is the proliferating layer. Whereas, iEGL express Pax6 alone and is devoid of Ki67 expression indicating that the iEGL is a post-mitotic layer. (**K**) Immunohistochemical analysis of Tlx3 and Ki67 show the co-localization of Tlx3 and Ki67 only in the EGL of posterior lobes. (**k’**) Further analysis of the magnified region indicate that Tlx3 and Ki67 co-localize in the oEGL (arrows) whereas, the iEGL expresses only Tlx3 (arrowheads) indicating that Tlx3 expression starts in the proliferating oEGL and is also expressed in the iEGL that are post-mitotic. We also see post- mitotic expression of Tlx3 in the IGL (arrowheads). (**k”**) Magnified area of the brain stem shows Tlx3 expression in post-mitotic neurons that are negative for Ki67. (**L**–**P**) Immunohistochemical analysis of posterior lobes of PN7 cerebellum showed co-expression of Tlx3 and DCX, only in post-mitotic iEGL and IGL indicating that Tlx3 expression starts in oEGL and these progenitors enter a post-mitotic stage. These progenitors then migrate into the IGL all the while maintaining the expression of Tlx3. (**Q**–**T**) Immunohistochemical analysis with vGlut1 and Tlx3 antibodies shows co-expression of vGlut1 with Tlx3 in the IGL. (**t’**) Magnified region shows co-localization of vGlut1 and Tlx3 in neurons of IGL. Image K is generated by stitching together multiple images using Photoshop software. Scale bar = 100 μm.

Further detailed analysis showed that Tlx3 is expressed in the region of the EGL that is proliferating. Tlx3 expression starts in cerebellum from E15 stage when almost all the cerebellar granule progenitors are proliferating and extend into the PN stages (Fig. 3E–H). We confirmed this by looking for the expression of the proliferating cell marker Ki67 along with Tlx3 in E16 cerebellum. Our results showed that ~50% of the Tlx3 positive cells were also positive for Ki67 confirming that Tlx3 is expressed in the proliferating progenitors (Fig. 5A–D,d’,E). This observation contradicts with the previous reports of Tlx3 expression being restricted to post-mitotic neurons of the spinal cord^1^.

As the development of cerebellum proceeds, cerebellar granule neurons are arranged into different layers based on the developmental stage. Here, the cerebellar granule neurons form separate layers demarcating the cerebellum into EGL, which contains only proliferating neurons and IGL that contains differentiated and mature neurons. The EGL can be further divided into an outer layer called outer EGL (oEGL) that contains only proliferating CGNs and the inner EGL (iEGL), which contains the post-mitotic CGNs that have migrated from oEGL^19^ (Fig. 5A). These layers were clearly visualized with Ki67 and Pax6 co-immunofluorescence in PN7 mouse cerebellum. Here, Ki67 and Pax6 label a clear boundary of proliferating CGNs in the oEGL, whereas the iEGL was negative for Ki67, but positive for Pax6 (Fig. 5G–J). These results confirm the fact that Pax6^+^/Ki67^−^ CGNs present in the iEGL are post-mitotic and have migrated from the proliferating oEGL.

To further confirm the expression of Tlx3 in proliferating oEGL, immunofluorescence analysis with Ki67 and Tlx3 was carried out in PN7 mouse cerebellums where the EGL has apparently developed. Our results show the expression of Ki67 in EGL of all the lobes whereas Tlx3 expression was limited only to the posterior lobes (Fig. 5K). To our surprise our results showed the expression of Tlx3 in both the oEGL and iEGL. Further detailed analysis showed a clear boundary of proliferating CGNs in the oEGL labeled by both Ki67 and Tlx3, whereas, the iEGL was negative for Ki67 but positive for Tlx3 alone (Fig. 5k’). Since the oEGL is the proliferating layer expressing Ki67, it is confirmed that Tlx3 expression begins in the proliferating progenitors and is also limited to the posterior lobes alone (Fig. 5k’). These results are by itself novel finding since Tlx3 is reported to be expressed exclusively in the post-mitotic progenitors of the spinal cord^1^. Interestingly we do see the expression of Tlx3 in the post-mitotic iEGL and cells of the brain stem (Fig. 5k”), which are Ki67^−^ and are in agreement with previous findings. BrdU immunostaining of PN1 mouse cerebellum was carried out after giving a BrdU pulse for 10 hours to further confirm the expression of Tlx3 in proliferating progenitors. These results supported the above observations, since the Tlx3 active cells in posterior oEGL were also BrdU positive (Fig. S4A,a’) whereas the Tlx3 positive cells in brain stem were negative for BrdU (Fig. S4a”). From the above results, it is evident that the Tlx3 expression begins in the proliferating prospective EGL of E15/16 cerebellum.

Even though Tlx3 expression begins in the mitotic stage of CGN development, its expression pertains till the CGNs differentiate and migrate to the IGL that consist of differentiated CGNs, UBCs as well as GABAergic interneurons formed from the ventricular zone. We carried out co-immunofluorescence analysis with Doublecortin (DCX) and Tlx3 to confirm whether the Tlx3 active cells migrate from the EGL to IGL. DCX labels all the migrating cells, and it was found that Tlx3 active cells in iEGL and IGL of posterior cerebellar lobes co-expressed DCX (Fig. 5L–P). To further substantiate the fact that Tlx3 active neurons in the IGL are glutamatergic neurons, we carried co-immunofluorescence analysis with vGlut1 and Tlx3 antibodies in PN7 mouse cerebellum. vGlut1 specifically labels the matured glutamatergic cerebellar granule neurons and our results showed that a majority of vGlut1 positive neurons in the IGL co-expressed Tlx3 (Fig. 5Q–T). These results confirmed the fact that Tlx3 expressing neurons in the IGL are the glutamatergic CGNs that have differentiated and migrated from the EGL. Our results also raised a very interesting question regarding the role played by Tlx3 specifically in the posterior lobes of the cerebellum. Prima facie both the anterior and posterior lobes look similar in its composition and architecture at PN7 stage but have compartmentalized expression of Tlx3. If that is the case then what would be Tlx3 specifically doing in the early stage of development (E16) in the prospective posterior lobe progenitors and PN7 posterior lobes? Many other genes such as Otx1, Otx2, Wnt-7b and Gli1 are shown to express in a compartmentalized pattern in the developing cerebellum. Most of these genes label the anterior, posterior or nodular lobes of the cerebellum specifically^20^. But the functional relevance of this compartmentally restricted expression pattern of genes is yet to be understood. Since Tlx3 also follows this particular expression pattern labeling the CGNs present in the posterior lobes of the cerebellum, we further wanted to analyze the possible downstream targets that would be promoted by Tlx3. These could be genes/regulators that are involved in imparting specific functional identity to the anterior and posterior lobes of the cerebellum.

### Tlx3 induces the expression of cholinergic receptor α3 specifically in posterior lobes of cerebellum

**Figure 6:**
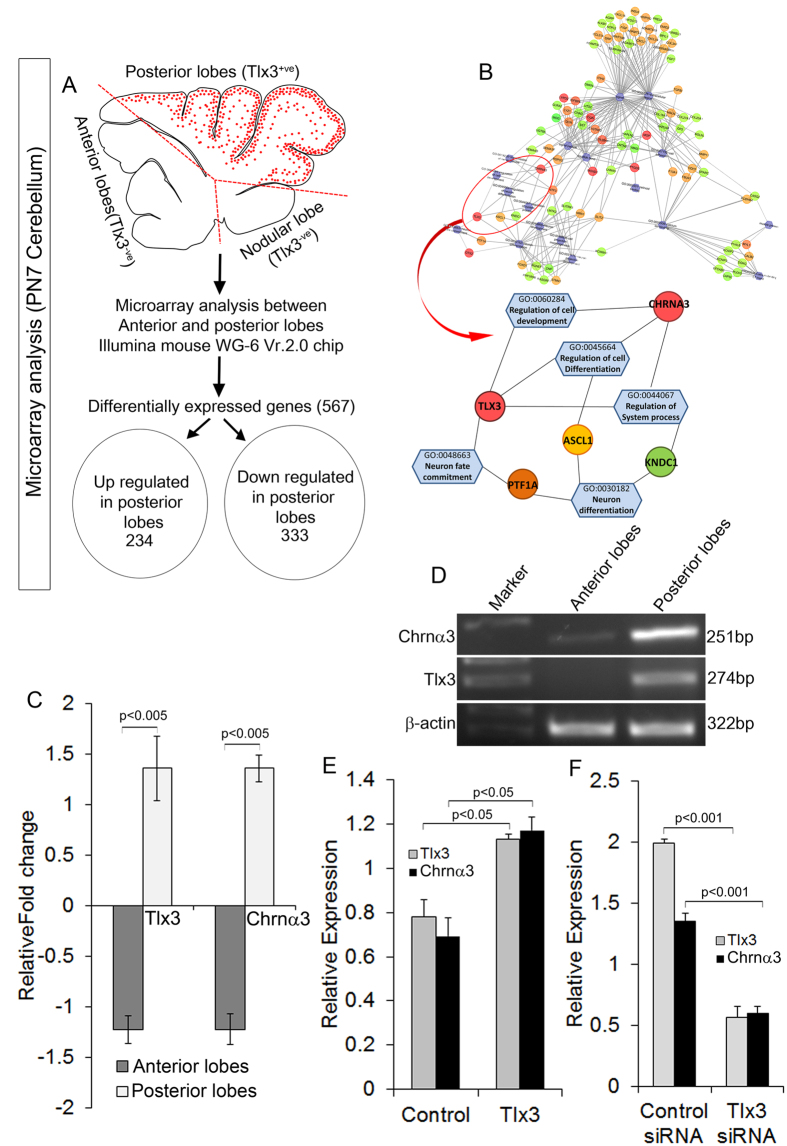
Microarray analysis of anterior and posterior regions of the cerebellum showed that Chrnα3 is up-regulated in the posterior cerebellum lobes. (**A**) Schematic showing the protocol used for microarray analysis that was carried out between anterior and posterior lobes of PN7 mouse cerebellum. Of the 567 genes differentially expressed with 1.5 fold change, 234 genes were up-regulated, and 333 genes were down-regulated in the posterior lobe compared to the anterior lobe. (**B**) Schematic of biological network created from the microarray data showed that Tlx3 is connected to Chrnα3 based upon the predicted biological functions. (**C**) Graph showing relative fold change of Tlx3 and Chrnα3 between anterior and posterior cerebellum. Both Tlx3 (p < 0.005) and Chrnα3 (p < 0.005) were up-regulated in posterior lobes compared to anterior lobes. (**D**) RT-PCR analysis of anterior and posterior cerebellum confirmed the microarray result since both Tlx3 and Chrnα3 was up-regulated in posterior compared to anterior cerebellum. (**E**) Real-time PCR analysis in PN7 cerebellar cultures with overexpression of Tlx3 show a significant increase (p < 0.05) in Chrnα3 expression. **(F**) Real-time PCR analysis in PN7 cerebellar cultures treated with Tlx3 siRNA showed a significant down regulation (p < 0.001) of Chrnα3 expression.

To understand this we dissected out the anterior and posterior lobes of PN7 mouse cerebellum and carried out microarray analysis in Illumina platform using the mouse WG-6 Vr.2.0 chip. Our results showed differential expression of various sets of transcription factors and receptors that were differentially expressed in posterior lobes. A total of 567 genes (234 up-regulated and 333 down-regulated) were identified to be differentially expressed with 1.2 fold difference in posterior compared to anterior cerebellum (Fig. 6A). Our results showed that Tlx3 expression was highly up regulated only in the posterior lobes as described earlier (Fig. 6C). This observation increased our confidence in our microarray and allowed us to proceed further. Functional annotation of the differentially expressed genes was retrieved using DAVID software, and a biological network was predicted. One of the predicted clusters linked to Chrnα3, a subunit of nicotinic acetylcholine receptors (Fig. 6B). Further analysis of microarray data showed that Chrnα3 was highly expressed only in the posterior lobes and correlated with the expression of Tlx3 (Fig. 6C). These results obtained through microarray analysis were confirmed by RT-PCR, which showed a similar higher expression of Tlx3 and Chrnα3 in the posterior lobes of cerebellum (Fig. 6D). Previous report by Gatta *et al*. using ChIP sequencing data in T-cell Leukemia samples has shown that Tlx3 can bind to Chrnα3^21^. Taking into account all these information, it now becomes apparent that Chrnα3 could be one of the possible downstream targets of Tlx3 in cerebellum. To substantiate Tlx3 and Chrnα3 interaction, we carried out perturbation experiments by overexpressing and down regulating Tlx3 using siRNA in PN7 cerebellar cultures and carried out real-time PCR analysis. Our results showed a significant increase (p < 0.05, Fig. 6E) in Chrnα3 expression upon overexpression of Tlx3 and a significant reduction (p < 0.001, Fig. 6F) in Chrnα3 expression when Tlx3 expression was down regulated. The dependence of Chrnα3 on Tlx3 was also demonstrated by down regulating Tlx3 using Tlx3 siRNA on cultured CGNs by immunocytochemical analysis. Our results showed a significant reduction (p < 0.001) in Chrnα3 expression in CGN cultures treated with Tlx3 siRNA compared with scrambled siRNA treated controls (Fig. S2R–T).

**Figure 7:**
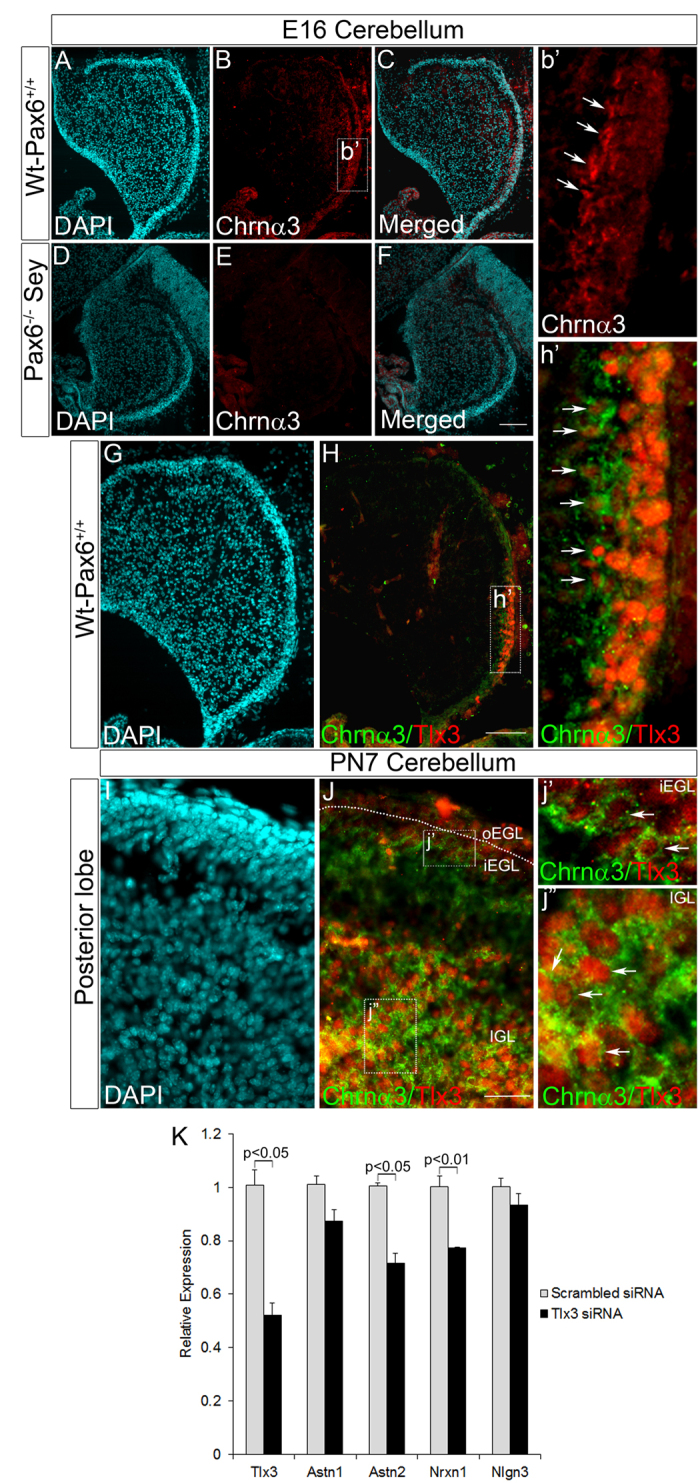
Pax6 through Tlx3 regulates Chrnα3 expression in posterior lobes of the cerebellum. (**A**–**C**) Immunohistochemical analysis of E16 Wt-Pax6^+/+^ cerebellum showed that Chrnα3 specifically labels EGL of the posterior region of cerebellum. (**b’**) Magnified region showed that Chrnα3 is expressed only in CGNs of prospective posterior region of the cerebellum. (**D**–**F**) Chrnα3 expression was entirely absent in Pax6^−/−^Sey cerebellum. (**G**–**H**) Immunohistochemical analysis of Tlx3 and Chrnα3 in E16 cerebellum showed that both these proteins are co-expressed specifically in the posterior EGL of cerebellum. (**h’**) Magnified region showed Chrnα3 co-expression with Tlx3 only in CGNs of prospective posterior region of the cerebellum. (**I**–**J**) Immunohistochemical analysis of Tlx3 and Chrnα3 in PN7 cerebellum showed that both these proteins co-express in iEGL and the expression is maintained till CGNs migrate to IGL. (**j’**) Magnified region of IGL showing cells co-expression Chrnα3 and Tlx3. (**K**) Real-time PCR analysis in PN7 cerebellar cultures treated with Tlx3 siRNA showed a significant down regulation in ASTN2 and NRXN1 (p < 0.05 and p < 0.01 respectively). Scale bar = 100 μm.

To further confirm our finding regarding Tlx3/Chrnα3 cascade, we carried out immunofluorescence analysis on E16 Wt-Pax6^+/+^ and Pax6^−/−^ Sey mouse cerebellum with Chrnα3 antibody. Here, we used the Pax6^−/−^ Sey mouse since we have already proved that Tlx3 expression is entirely absent in this model (Fig. 4J–L). Consistent with our prediction we observed Chrnα3 expression in Wt-Pax6^+/+^ cerebellum (Fig. 7A–C,b’) and was entirely absent in Pax6^−/−^ Sey cerebellum (Fig. 7D–F). The expression of Chrnα3 in Wt-Pax6^+/+^ cerebellum was restricted to the region of prospective posterior lobes. Next, to show the co-localization of Tlx3 and Chrnα3, we subjected E16 Wt-Pax6^+/+^ cerebellum to co-immunofluorescence analysis with Tlx3 and Chrnα3 antibodies. Our results showed that Chrnα3 and Tlx3 co-localized only in the post-mitotic EGL and in the cells that are migrating into the prospective IGL. These results indicate that Tlx3 expression precedes the appearance of Chrnα3 (Fig. 7G,H,h’). We extended the co-localization studies to PN7 cerebellum and found co-localization of Tlx3 and Chrnα3 only in the iEGL and IGL of posterior lobes and not in the oEGL (Fig. 7I,J).

**Figure 8:**
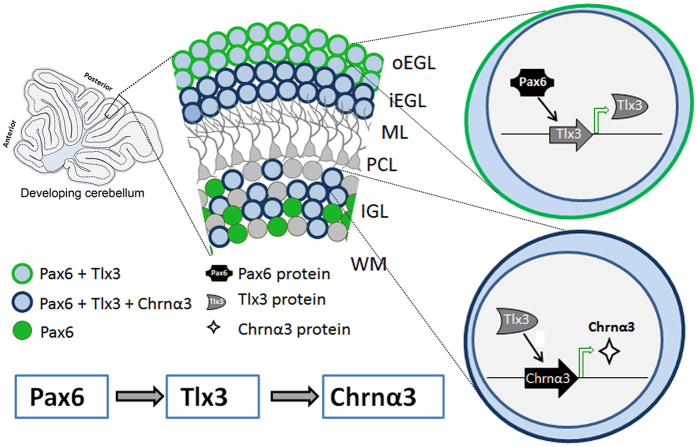
Schematic depicting the expression of Chrnα3 in posterior lobes of developing cerebellum through Pax6-Tlx3 regulation. Pax6 induces the expression of Tlx3 in proliferating oEGL. These progenitors further enter a post-mitotic state and migrate into the iEGL all the while maintaining Tlx3 expression. Here, Tlx3 induces the expression of cholinergic receptor subunit Chrnα3 and these progenitors further migrate into the IGL and differentiate into glutamatergic neurons with Chrnα3 expression. The Pax6-Tlx3-Chrnα3 interaction is restricted and compartmentalized only to the posterior lobes of the developing cerebellum.

Therefore, it appears that the Tlx3 can induce expression of Chrnα3 specifically in the glutamatergic neurons of posterior cerebellum and distinguishes them as a distinct subpopulation compared to the other glutamatergic neurons of the anterior lobes (Fig. 8). Previous studies have shown that Chrnα3 is expressed in cerebellar granule neurons and is down regulated in autism spectrum disorders^12^. Therefore, these cholinergic receptor complexes having Chrnα3, as a sub-unit may be responsible for receiving specific signals from unique innervations connecting to the posterior CGNs^22^. These could be the reasons for the compartmentalization of gene expression, which makes the CGNs of the posterior lobes physiologically or functionally distinct. Since Chrnα3 is linked with ASD, we extended our study to check the possibility that Tlx3 could play a role in ASD by analyzing ASD candidate genes (down-regulated in ASD) such as Astrotactin1 (Astn1), Astrotactin2 (Astn2), Neurexin1 (Nrxn1) and Neuroligin3 (Nlgn3) which play an important role in neuronal migration and synaptic connection formation^23^. We further down-regulated Tlx3 using Tlx3 siRNA in PN7 CGN culture and carried out RT-PCR analysis. Out of the four genes analyzed, we could observe a significant reduction in Astn2 and Nrxn1 (p < 0.05 and p < 0.01 respectively, Fig. 7K), however we could not find a significant reduction in Astn1 and Nlgn3. These results indicate that there could be a possible association of Tlx3 with Astn2 and Nrxn1, either directly or indirectly linking them to ASD.

## Discussion

In this report, we have described an additional role for Tlx3 that is activated by Pax6 in the posterior lobes of the cerebellum and the resultant expression of Chrnα3. We now know that Tlx3 is required for induction of Chrnα3 in the posterior lobes, but the reason for the appearance of Tlx3 only in the posterior lobes is beyond the scope of this study. We assume that this may be due to specific epigenetic modifications happening to the Tlx3 promoter in the anterior lobes. Functionally, it appears that the posterior lobes receive most of the inputs from the brainstem (i.e., reticular formation and inferior olivary nucleus) and cerebral cortex^24^. Further studies are needed to unravel the exact mechanism for the compartmentalized expression pattern of Tlx3 in cerebellum.

We also show the expression of Tlx3 in CGNs of proliferating oEGL, which by itself is a novel finding since Tlx3 till now, is known to express only in post-mitotic neurons of spinal cord and brain stem. These results suggest that Tlx3 may have a unique role in proliferating CGNs. It’s possible that Tlx3 expression observed in proliferating CGNs may be required to shift them towards differentiation, but the exact function for its expression during proliferation needs to be explored further. We do see expression of Tlx3 in post-mitotic brain stem and spinal cord neurons which remains unaffected in Pax6^−/−^ Sey mouse revealing that Pax6 tightly regulates the expression of Tlx3 in a compartmentalized manner specifically in the cerebellum. Interpretation of our data also revealed that proliferating progenitors expressing Tlx3 in the oEGL enter a post-mitotic stage by migrating into the iEGL and then to IGL all the while retaining their Tlx3 expression. Once in the iEGL, Tlx3 induces the expression of Chrnα3 in the differentiating excitatory neuron (Fig. 8). It appears that Chrnα3, which is a subunit of cholinergic receptors, may be required for the generation of a subset of cholinergic receptors expressing neurons in the posterior cerebellum. This compartmentalized expression pattern of cholinergic receptors may be necessary to direct specific neuronal innervations in the posterior cerebellum during development so that unique synaptic connections receiving specific signals that control particular functional aspects are formed. Structurally both the anterior and posterior lobes look similar after development with respect to the excitatory neurons. The intriguing question that now arises is why Pax6 should induce expression of Tlx3 only in the posterior lobes in a compartmentalized manner and the role of Tlx3 in proliferation of EGL progenitors that warrants further investigation. We would also like to relate our findings to the observation that Chrnα3 expression is significantly reduced in cerebellar granule cells of patients with autistic spectral disorders^12^. Several studies from the past have prominently shown cerebellum as the region of interest in the pathophysiology of ASD. This could be due to impaired regulation of genes that are involved in the neuronal migration and neurodevelopment. *Astn2*, expressed by the cells in the EGL, IGL, molecular layer and the Purkinje cells, is essential for the cerebellar granule cell migration^25^. Mutation in *Astn2* has been reported in autism^23^ and other neuropsychiatric disorders^26^,^27^. In addition to this, *Astn2* regulates the expression of *Astn1* on the cell surface to aid in the neuron-glial mediated neuronal migration^25^. Similarly, neurexins (Nrxn) are the receptors of neuroligins (Nlgn) which regulate the excitatory synaptic function. Mice lacking Nlgn1, Nlgn2 and Nlgn3 die at birth due to respiratory failure^28^ which is similar to the observations seen in Tlx3 knock-outs^4^. There are several other mutations in the *Nrxn1* and *Nlgn3* which have been reported in autistic patients^29^. Our results show that when Tlx3 is down-regulated, expression levels of *Astn2* and *Nrxn1* are also reduced indicating that Tlx3 could possibly have a role in neuronal migration and synaptic functions (Fig. 7K). The observed regulation of *Chrn*α3, *Astn2* and *Nrxn1* by Tlx3 could be direct or indirect through other factors which need to be explored further. Since Tlx3 expression in cerebellum is only during the early development, any deregulation of Tlx3 during this period could alter the cholinergic receptor expression, neuronal migration and synaptic connections. All these parameters are found to be similarly altered in ASD patients and could be linked to the changes in early development. We speculate that these alterations in the above parameters during early cerebellar development could manifest as ASD during adolescent stage. We assume that Tlx3 may be a part of this complex gene regulatory network that is disrupted in neurodevelopmental disorders such as ASD which definitely needs to be investigated further. Therefore, overall we have convincingly demonstrated the activation of Tlx3 by Pax6 in developing posterior cerebellum, a new role for Tlx3 in promoting compartmentalized expression of *Chrnα3* in cerebellum and possible involvement in regulation of the genes involved in formation of synaptic connections and neuronal migration.

## Experimental Procedures

### Microarray analysis

The anterior and posterior lobes of PN7 mouse cerebellum were dissected out separately in RNAse free conditions. RNA isolation was carried out as previously as described using Tri Reagent (Sigma Aldrich)^30^. Two replicates of each sample were used for analysis, and the RNA integrity was verified using Agilent Bioanalyzer. Microarray hybridization, data acquisition, and analysis were outsourced to Bionivid Technology Pvt. Ltd, Bangalore, India (http://www.bionivid.com). Microarray analysis between anterior and posterior lobes was carried out in an Illumina platform using mouseWG-6_V2_0_R3_11278593_A chip with the threshold set as 1 and log base as 2. Genes that had a fold change >1.2 and a p-value < 0.05 were considered as differentially expressed. Whole mouse Genome was used as the reference group and the gene ontology and pathway analysis was performed using GO-Elite software to identify overrepresented GO-classes and pathways were compared with the mouse whole genome. Statistical significance was calculated with a standard hyper geometric distribution corrected by a Benjamini Yekutelli correction for multiple testing, that takes into account the dependency among the GO categories. The minimal length of considered GO-paths was 2. The functional annotation of the differentially expressed genes were carried out using DAVID Bioinformatics Resources 6.7 (http://david.abcc.ncifcrf.gov) online software and the biological network connecting the specific genes were generated for further downstream analysis. The microarray data has been submitted to Gene Expression Omnibus (GEO accession No. GSE82097).

### Generation of specific plasmid constructs

Mouse Tlx3 promoter (1000bp) was PCR amplified from genomic DNA of mouse brain using specific primers (listed in Table S1) and was initially cloned into PCRII TA cloning system. The pTlx3-luc vector was constructed by directionally cloning KpnI/XbaI digested fragment of 1000bp size into promoter less pGL3 basic vector digested with KpnI/NheI and was used for luciferase analysis. Amplification of mouse Tlx3 promoter with mutated Pax6 binding site was carried out with specific primers (Table S1) using pGL3-mTlx3 as template. The mutated promoter sequence was amplified using Advantage GC cDNA polymerase Kit (Clontech). pTlx3-EGFP construct used to check the activity of Tlx3 promoter in HeLa cells was made as described earlier^31^. pCAGIG-Pax6 and pCAGIG-Pax6Δ286 were produced by amplifying the Pax6 and Pax6Δ286 regions from pBI-EGFP-Pax6 and pBI-EGFP-Pax6Δ286 respectively (kind gifts from Dr. Elizabeth Fini, The University of Miami, Florida) using primers listed in Table S1. The amplified region was cloned using *XhoI* and *Not1* restriction sites, which were introduced into the forward and reverse primers respectively. The pCAGIG-Pax6 and pCAGIG-Pax6Δ286 plasmids were used for up-regulating and down-regulating Pax6 respectively and were used for luciferase and RT-PCR analysis. Pax6 siRNA was used to down regulate the endogenous Pax6 (Sigma Aldrich).

### Generation of Pax6^−/−^Sey Knock Out mouse

Pax6^−/−^Sey mouse (Pax6 Knock Out) were generated by crossing heterozygous Pax6^+/−^Sey x Pax6^+/−^Sey mice and E16 embryos for the experiments were provided by Dr. Shubha Tole, TIFR, Mumbai (Pax6^+/−^Sey mice were originally obtained from Prof. Anastassia Stoykova, Max Plank Institute for Biophysical Chemistry, Germany). Pax6^−/−^Sey embryos were identified by the typical eyeless morphology. Due to the presence of a point mutation in Pax6^−/−^Sey strain, even though the transcription is not affected it leads to the production of a truncated nonfunctional protein^32^. Brain samples of Wt-Pax6^+/+^ and Pax6^−/−^Sey mice were collected at embryonic day 16 and were analyzed for expression of Pax6, Tlx3 and Chrnα3.

### HeLa and primary Cerebellar Granule Neuron culture

We used HeLa cells (Cells sourced from ATCC and were tested for contamination) initially to analyze the effect of Pax6 on Tlx3 since HeLa cells have constitutive expression of Tlx3^33^. The cells were expanded in a medium containing DMEM (Invitrogen) with 10% FBS (Sigma Aldrich) at 37 °C with 5% CO2 and were trypsinized at about 70–80% confluency with 0.05% Trypsin. The cells were grown in 24-well plates for luciferase assay and RT-PCR analysis. pTlx3-luc, pCAGIG-Pax6 and pCAGIG-Pax6Δ286 were transfected using Lipofectamine 2000 (Invitrogen) as per the manufacturer’s protocol, and luciferase assay and RT-PCR analysis were carried out.

Primary culture of granule neurons was prepared from 7 day old mouse pup cerebellum according to the protocol of Lee *et al*.^34^. The protocol for the use of animals was approved by the Institutional animal ethics committee (IAEC) of Rajiv Gandhi Center for Biotechnology (RGCB) and the methods were carried out in accordance with the approved guidelines. Briefly, the cerebellum of PN7 mouse pups were dissected out under sterile conditions and the tissue was dissociated using 0.05% Trypsin in the presence of DNAase (Ambion). Thereafter, cells were collected by centrifugation and re-suspended in Neurobasal medium (Gibco) containing B27 supplement (Gibco), 10% Fetal Bovine Serum (Sigma Aldrich), 1X glutamax (Gibco), 430 μM glucose and 20 mM KCl. Cells were seeded onto 24 well plates, coated with 10  μg/ml of poly-D-lysine (Sigma Aldrich) and 5 μg/ml laminin (BD Biosciences), at a density of 2 × 10^6^ cells/well. Primary cerebellar cultures were transfected with pCAGIG-Pax6, Pax6 siRNA (Sigma Aldrich), scrambled siRNA and Tlx3 siRNA (Dharmacon) by electroporation using Neon Electroporator (Invitrogen) according to manufacturer’s protocol and processed for RT-PCR analysis.

### Semi-quantitative and real-time PCR analysis

For RT-PCR analysis of HeLa cells, total RNA was isolated using Qiagen RNA easy kit (Qiagen). The isolated RNA was treated with DNAse to avoid any DNA contamination. Equal amount of RNA was reverse transcribed into cDNA using superscript RT as described previously^31^. cDNA of different samples was normalized using β-actin, and the specific products were amplified using specific primers (Table S1) on an ABI Veriti thermocycler (Applied Biosystems, USA).

SYBR green based real-time PCR was used to analyze the expression of specific genes in cerebellar granule neuron cultures (Table S1). ~1μg of total RNA was converted to cDNA using Random hexamers (Promega) and superscript RT-II (Invitrogen). The expression level of specific genes of interest was analyzed using Takara SYBR green mix with β-actin as internal control. The real-time analyzes were done by 2^−ΔΔCt^ method using SDS 2.1 software (ABI)^35^.

### Dual-luciferase assay

HeLa cells in 24-well plates were transfected with respective plasmid using Lipofectamine 2000 (Invitrogen) in OPTI-MEM medium as per the manufacturer’s instructions. Each transfection was done in triplicate and for co-transfections, the DNA concentration in each tube was normalized by adding control pCAGIG vector. After 6 h of transfection, the medium was replaced with fresh growth medium and incubated at 37 °C for an additional 48 h. Cell lysis followed it as per the manufacturer’s protocol (Promega) and luciferase assay was performed in a luminometer (TD 20/20 Luminometer) with dual luciferase mode. Each experiment was done in triplicate, and the firefly luciferase values were normalized using Renilla luciferase values and a graph was plotted with these normalized values^31^.

### Immunofluorescence analysis

Immunocytochemical analysis was carried out on 5-day differentiated cerebellar granule neuron cultures. The cells were washed once in 1X PBS and fixed in 4% paraformaldehyde for 15 min at 4 °C followed by blocking in 5% NGS (Normal Goat Serum; Sigma-Aldrich). The cells were permeabilized with 0.4% for nuclear and 0.2% for cytoplasmic antibodies with Triton-X100.

For immunohistochemical analysis, mouse brains were obtained at different developmental stages (E14-PN7) and fixed in 4% paraformaldehyde overnight at 4 °C. The fixed whole brains were dehydrated in 30% sucrose and embedded in OCT for cryosectioning. 12 μm thick cryo-sections were cut, permeabilized and further used for immunofluorescence analysis. The primary antibodies used were Pax6 (Chemicon-AB2237, 1:200), Tlx3 (1:2,000, gift from Dr. Carmen Birchmeier, Germany), vGlut1 (Chemicon-MAB5502, 1:200), GABAα6 (Chemicon- AB5610, 1:200), β-III tubulin (1:200, Chemicon-MAB5564) and Chrnα3 (Santacruz-sc-5590, 1:10). Sections were examined for epifluorescence following incubation with appropriate secondary antibody conjugated to Cy3/Alexa Fluor488. To rule out any possibility of Tlx3 antibody picking up any cell non-specifically we induced Tlx3 expression in SVZ of cortex (Tlx3 expression is entirely absent in cortex during development) of E14 embryos by *In utero* electroporation of Tlx3 expression vector that also expresses EGFP. These embryos were sectioned at E16 stage and subjected to immunofluorescence analysis with Tlx3 antibody that co-localized only with Tlx3 expressing (EGFP expressing) cells (Fig. S4B–E).

For BrdU immunostaining, the brain samples were collected after pulsing with 100 mg/kg BrdU for 10 hours. The DNA was denatured by incubating tissue sections in 2N HCl for 45 min at 37 °C, followed by a 10 min incubation in 0.1 M boric acid in 1X PBS, pH 8.5. BrdU was detected with anti-BrdU antibody (1:200, Abcam-ab6326) and nucleus of cells was co-localized using DAPI. Fluorescence was analyzed using an upright fluorescent microscope (Olympus BX61), and images were captured using a cooled CCD camera (Andor 885).

### Statistical analysis

Statistical significance between the groups was calculated by independent Student’s *t*-test assuming equal variance. Values with p < 0.05 were considered as statistically significant.

## Additional Information

**How to cite this article**: Divya, T. S. *et al*. Regulation of Tlx3 by Pax6 is required for the restricted expression of Chrnα3 in Cerebellar Granule Neuron progenitors during development. *Sci. Rep.* **6**, 30337; doi: 10.1038/srep30337 (2016).

## Supplementary Material

Supplementary Information
