# Supplementary material for: Regulation of Tlx3 by Pax6 is required for the restricted expression of Chrnα3 in Cerebellar Granule Neuron progenitors during development
Source: Sci Rep. 2016 Jul 25;6:30337. doi: 10.1038/srep30337 (PMC4959012; doi:10.1038/srep30337)
Supplement: Supplementary Information [file srep30337-s1.pdf]

Supplementary Information

Regulation of Tlx3 by Pax6 is required for the restricted expression of Chrnα3 in Cerebellar Granule Neuron progenitors during development

Thulasi Sheela Divya[1], Soundararajan Lalitha[1], Surendran Parvathy[1], Chandramohan Subashini[1], Rajendran Sanalkumar[1,3], Sivadasan Bindu Dhanesh[1], Vazhanthodi Abdul Rasheed[1], Mundackal Sivaraman Divya[1,4], Shubha Tole[2] & Jackson James[1]*

[1] *Neuro Stem Cell Biology Laboratory, Neurobiology Division, Rajiv Gandhi Centre for Biotechnology, Thiruvananthapuram, Kerala-695 014, India*

[2] *Department of Biological Sciences, Tata Institute of Fundamental Research, Mumbai-400005, India*

Fig. S1

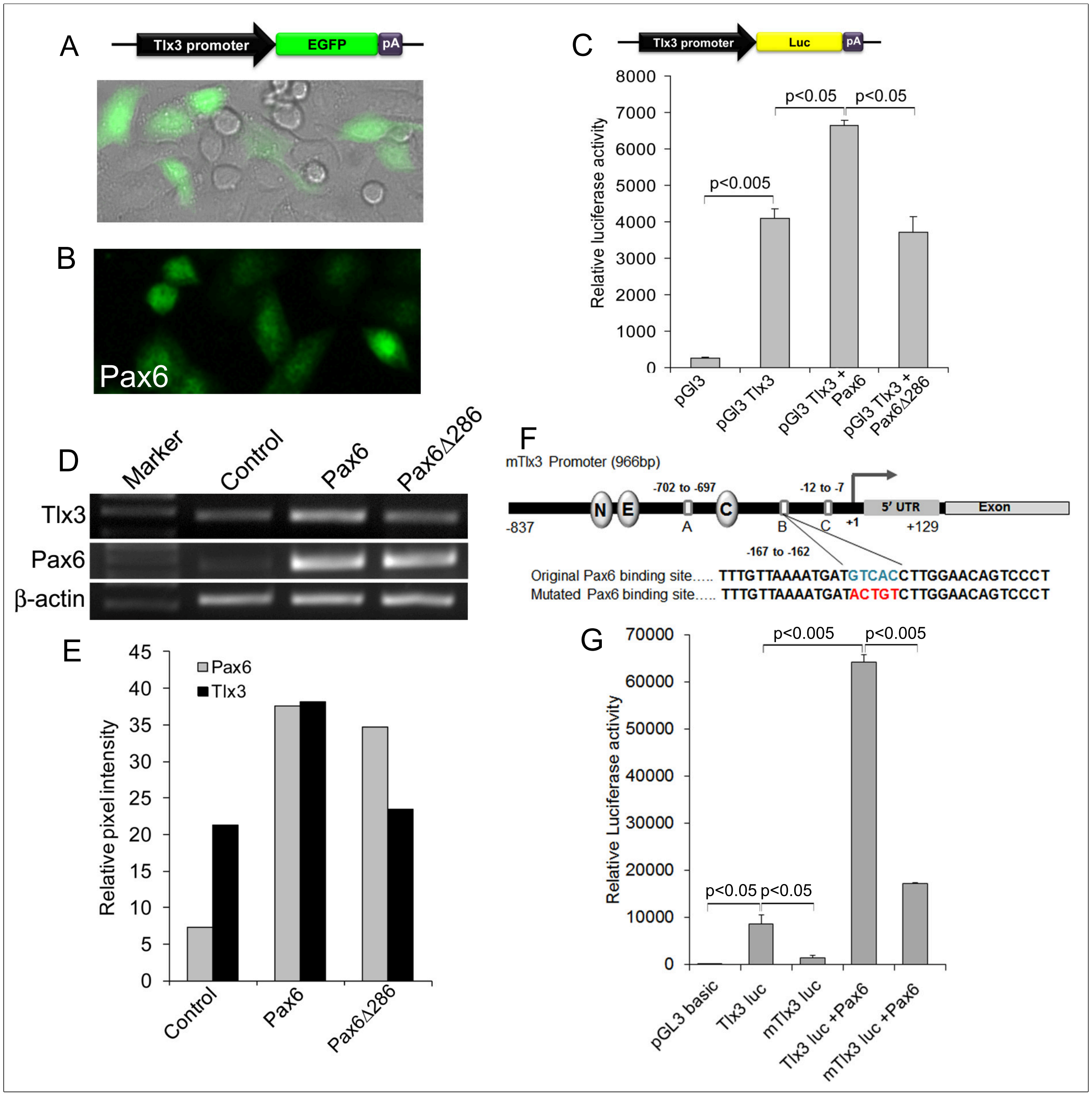
A
Tlx3 promoter
EGFP
pA
B
Pax6
C
Tlx3 promoter
Luc
pA
Relative luciferase activity
8000
7000
6000
5000
4000
3000
2000
1000
0
p<0.005
p<0.05
p<0.05
pGl3
pGl3 Tlx3
pGl3 Tlx3 + Pax6
pGl3 Tlx3 + Pax6Δ286
D
Marker
Control
Pax6
Pax6Δ286
Tlx3
Pax6
β-actin
E
Relative pixel intensity
45
40
35
30
25
20
15
10
5
0
Pax6
Tlx3
Control
Pax6
Pax6Δ286
F
mTlx3 Promoter (966bp)
-702 to -697
-12 to -7
N
E
C
A
B
C
+1
5' UTR
Exon
-837
+129
-167 to -162
Original Pax6 binding site..... TTTGTTAAAATGATGTCACCTTGGAACAGTCCCT
Mutated Pax6 binding site..... TTTGTTAAAATGATACTGTCTTGGAACAGTCCCT
G
Relative Luciferase activity
70000
60000
50000
40000
30000
20000
10000
0
p<0.005
p<0.005
p<0.05
p<0.05
pGL3 basic
Tlx3 luc
mTlx3 luc
Tlx3 luc +Pax6
mTlx3 luc +Pax6

Fig.S2

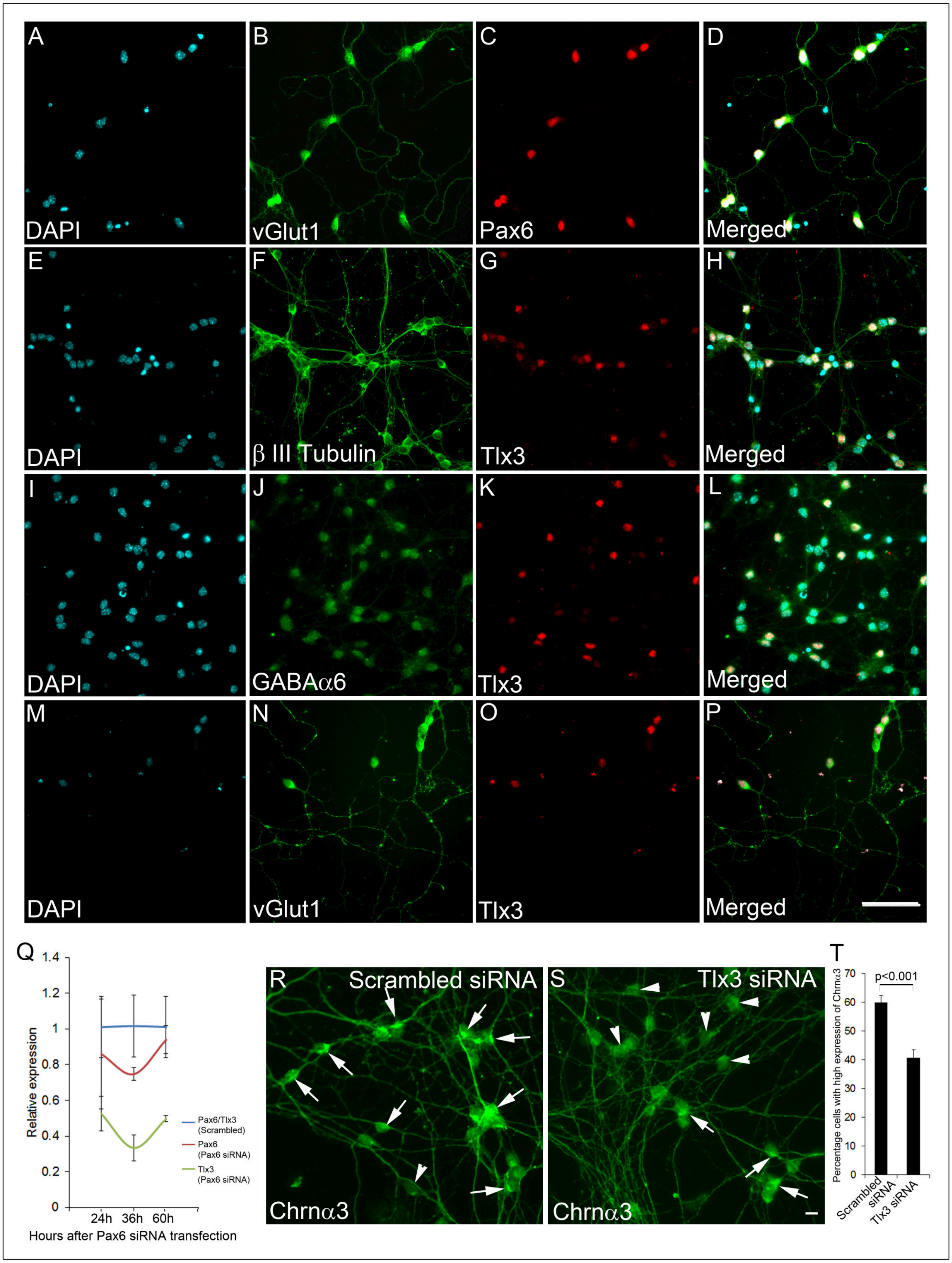
A
DAPI
B
vGlut1
C
Pax6
D
Merged
E
DAPI
F
β III Tubulin
G
Tlx3
H
Merged
I
DAPI
J
GABAα6
K
Tlx3
L
Merged
M
DAPI
N
vGlut1
O
Tlx3
P
Merged
Q
Relative expression
Hours after Pax6 siRNA transfection
24h
36h
60h
Pax6/Tlx3 (Scrambled)
Pax6 (Pax6 siRNA)
Tlx3 (Pax6 siRNA)
R
Scrambled siRNA
Chrnα3
S
Tlx3 siRNA
Chrnα3
T
Percentage cells with high expression of Chrnα3
p<0.001
Scrambled siRNA
Tlx3 siRNA

Fig. S3

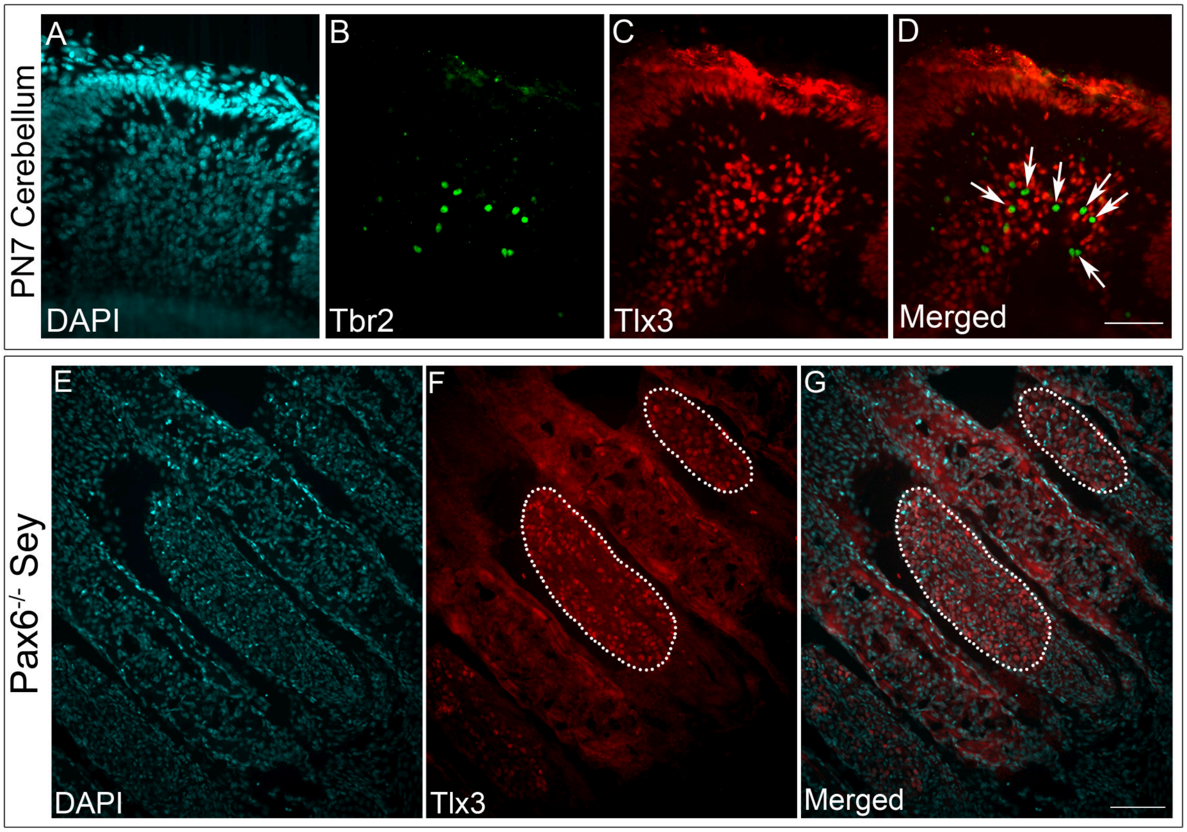

PN7 Cerebellum
A
DAPI
B
Tbr2
C
Tlx3
D
Merged
Pax6-/- Sey
E
DAPI
F
Tlx3
G
Merged

Fig. S4

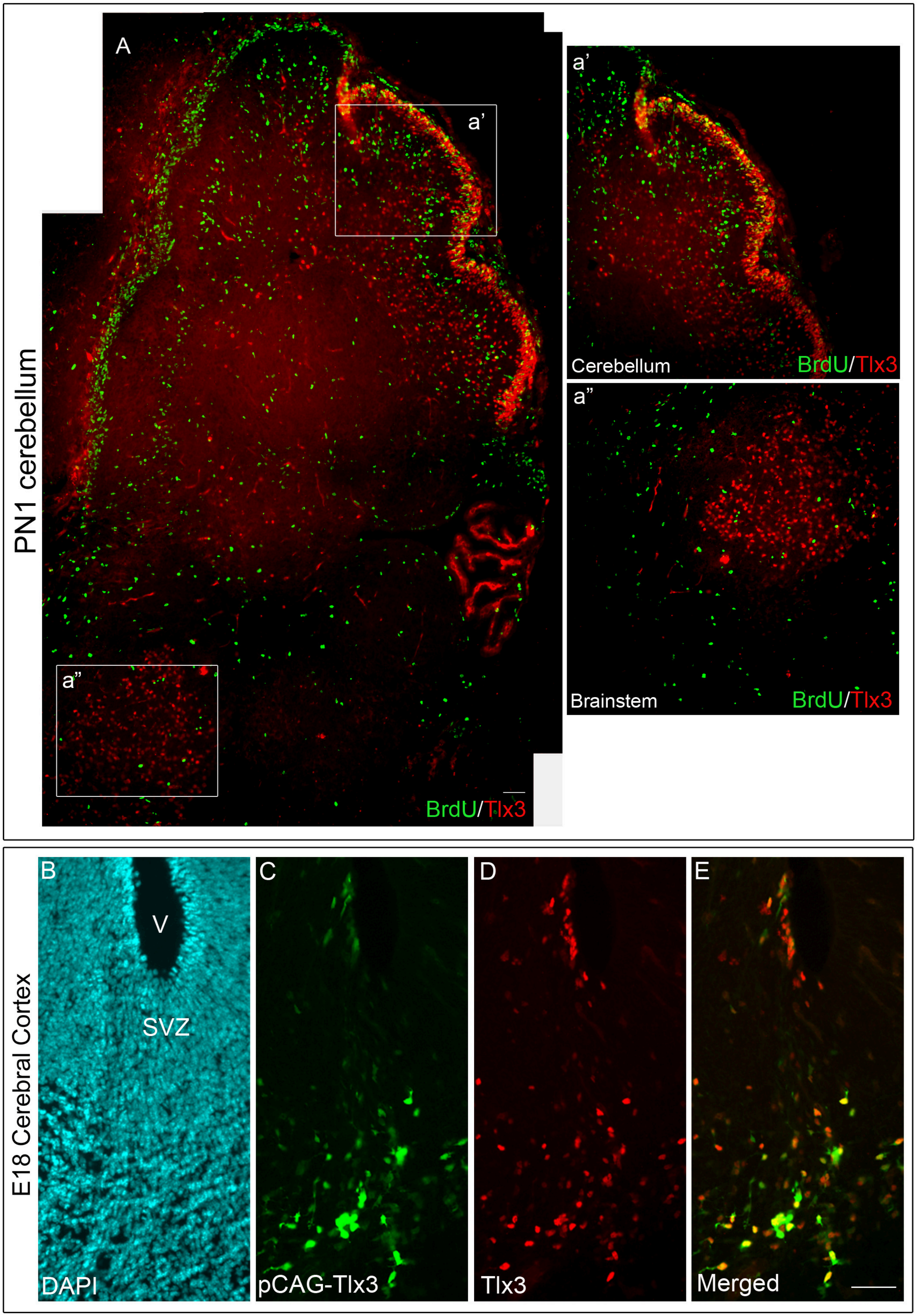
PN1 cerebellum
A
a'
a"
BrdU/Tlx3
a'
Cerebellum
BrdU/Tlx3
a"
Brainstem
BrdU/Tlx3
E18 Cerebral Cortex
B
V
SVZ
DAPI
C
pCAG-Tlx3
D
Tlx3
E
Merged

Table S1: List of Primers used

Gene Name	Primer sequence (5'-3')	Ann temp. (^{0}C)	Product size (bp)
**β -actin**	F- AGACTTCGAGCAGGAGATG<br>R- CTTGATCTTCATGGTGCTAGG	56	322
**mTlx3 promoter amplification**	F- CCTCGGAGGGGTCCCAAACCAGCGACC<br>R- CCTGGGCGGGCGGAAG	56	966
**Tlx3**	F-GCGCATCGGCCACCCCTACCAGA<br>R- CCGCTCCGCCTCCCGCTCCTC	56	274
**Pax6**	F-GCAACACTCCTAGTCACAT<br>R- TGTAGGTATCATAACTCCG	56	282
**Chrnα3**	F- AAACCTGAACTGCTTCAGC<br>R- TGGCTACATTCTGTGCTTTC	56	251
**Astn1**	F-TCCCACTGCCAAACAGGATG<br>R -CTCTTCTGGAGCATGGGTTAGG	60	225
**Astn2**	F- CTTGGCCTTACACAACACTG<br>R- GGACAACATTGCTTCTTCCC	60	230
**Nrxn1**	F-TCAACACACTACGCACTCCC<br>R- CAGTGCTCTGAATTTCCGCC	56	225
**Nlgn3**	F-ACTCCTGGTTGAGAACCCTC<br>R- GGGACCCAACTGTAATGCTG	60	225
**Pax6 mutation**	F- GTTTGTTAAAATGATACTGTCTTGGAACAGTCCCT<br>R- AGGGACTGTTCCAAGACAGTATCATTTTAACAAAC	-	-
**Pax6 CDS amplification**	F- CTCGAGATGCAGAACAGTCACAGCGGA<br>R- GCGGCCGCTTACTGTAATCGAGGCCAGTA	56	1311
**Pax6 Δ286 CDS amplification**	F- CTCGAGATGCAGAACAGTCACAGCGGA<br>R- GCGGCCGCAGGAATGTGACTAGGAGTGTTGCT	56	900

Supplemental Information

Fig. S1: Pax6 up-regulates Tlx3 expression in HeLa cells.

A) Schematic of Tlx3 promoter-driven EGFP construct transfected into HeLa cells to confirm Tlx3 promoter activity by analyzing EGFP expression. **B)** Immunocytochemical analysis showed that majority of HeLa cells was positive for Pax6. **C)** Schematic represents Tlx3 promoter luciferase construct in which luciferase reporter is controlled by the activity of Tlx3 promoter. Graph depicts Luciferase activity in HeLa cells after transient transfection with Tlx3-luc construct that showed a significant increase ($p<0.005$) in luciferase activity compared to control pGl3 basic. The activity was further significantly up-regulated ($p<0.05$) with Pax6 was over-expression. Luciferase activity in HeLa cells after transient transfection with mutated Tlx3-luc construct showed a significant reduction ($p<0.05$) compared to Tlx3-luc construct. **D**) RT-PCR analysis showed that Pax6 could up-regulate Tlx3 expression in Hela cells. **E**) Pixel intensity graph of the data obtained from RT-PCR analysis. **F)** Schematic depicting 1kb Tlx3 promoter with mutated Pax6 binding site. **G)** Luciferase activity in HeLa cells after transient transfection with mutated Tlx3-luc construct (mTlx3-luc) showed a significant reduction ($p<0.05$) compared to the Tlx3-luc construct. Over expression of Pax6 along with Tlx3-Luc construct significantly enhanced ($p<0.005$) the promoter activity but it was significantly reduced ($p<0.005$) when Pax6 was transfected along with mutated mTlx3-Luc construct. Scale bar= 25μm

Fig. S2: Characterization of cerebellar granule neurons *in vitro*.

A-D) Immunocytochemical analysis showed that Pax6 positive CGNs co-expressed vGlut1, which specifically labels glutamatergic neurons. Tlx3 positive CGNs also co-expressed β III tubulin, a neuronal marker (**E-H**), CGN specific marker GABAα6 (**I-L**) and glutamatergic

neuron marker vGlut1 (**M-P**) which proved that the *in vitro* culture generated from PN7 mouse cerebellum would be suitable for further *in vitro* studies. **Q)** The specificity of Pax6 siRNA was confirmed by checking the down regulation of Pax6 and Tlx3 by real-time RT-PCR analysis at different time intervals (24h, 36h and 60h) after transfection (in PN7 CGN culture). Graph indicates a drastic reduction in Pax6 expression and corresponding Tlx3 expression by 36h after Pax6 siRNA transfection when compared to the scramble siRNA treated CGNs. Both Pax6 and Tlx3 expression pattern showed a reversing trend by 60h after transfection. **R&S)** Immunocytochemical analysis of CGNs transfected with Tlx3 siRNA showed a significant reduction in Chrnα3 expression. Arrows indicate cells with normal expression of Chrnα3. Arrowhead indicates cells with reduced Chrnα3 expression. **T)** Graph indicates a significant reduction ($p<0.001$) in percentage of cells with high expression of Chrnα3 upon Tlx3 siRNA transfection compared to control. Scale bar= 25µm.

Fig. S3: Tbr2^{+ive} and Tlx3^{-ive} cells in the IGL of posterior lobes represent the unipolar brush cells (UBCs) in the cerebellum.

A-D) Immunohistochemical analysis of PN7 mouse cerebellum shows Tbr2^{+ive} and Tlx3^{-ive} UBCs that are present in the IGL in addition to the differentiating CGNs. Scale bar= 100µm.

E-G) Immunohistochemical analysis of E16 Pax6$^{-/-}$Sey mouse spinal cord proved that Tlx3 expression is not dependent on Pax6 expression. Scale bar= 100µm.

Fig. S4: Proliferation of Tlx3 expressing progenitors was confirmed with BrdU incorporation and immunohistochemical analysis of *in utero* electroporated cerebral cortex confirms specificity of Tlx3 antibody.

A) Immunohistochemical analysis of PN1 mouse cerebellum showed BrdU incorporation in Tlx3^{+ive} mitotic CGN progenitors. **a'**) Magnified region of posterior cerebellum showing co-localization of BrdU and Tlx3 in EGL. **a"**) Magnified region of the brain stem showing Tlx3+ive cells without BrdU incorporation. Image A is generated by stitching together multiple images using Photoshop software. Scale bar A= 100µm. **B-E**) E18 cerebral cortex that were *in utero* electroporated with pCAG-Tlx3 expression construct and were probed with Tlx3 antibody to confirm the specificity of the antibody used for other experiments.

Table S1: List of Primers used
